# Rationally designed hierarchical porous CNFs/Co_3_O_4_ nanofiber-based anode for realizing high lithium ion storage†

**DOI:** 10.1039/c8ra06307a

**Published:** 2018-08-31

**Authors:** He Wang, Yan Song, Yanwei Li, Mengwei Wang, Qianli Ma, Wensheng Yu, Dan Li, Xiangting Dong, Jinxian Wang, Guixia Liu

**Affiliations:** School of Chemistry and Environmental Engineering, Changchun University of Science and Technology Changchun 130022 China xtdong@cust.edu.cn +86-0431-85383815 +86-0431-85582574

## Abstract

To achieve a high power density of lithium-ion batteries, it is essential to develop anode materials with high capacity and excellent stability. Cobalt oxide (Co_3_O_4_) is a prospective anode material on account of its high energy density. However, the poor electrical conductivity and volumetric changes of the active material induce a dramatic decrease in capacity during cycling. Herein, a hierarchical porous hybrid nanofiber of ZIF-derived Co_3_O_4_ and continuous carbon nanofibers (CNFs) is rationally constructed and utilized as an anode material for lithium-ion batteries. The PAN/ZIF-67 heterostructure composite nanofibers were first synthesized using electrospinning technology followed by the *in situ* growth method, and then the CNFs/Co_3_O_4_ nanofibers were obtained by subsequent multi-step thermal treatment. The continuous porous conductive carbon backbone not only effectively provides a channel to expedite lithium ion diffusion and electrode transfer, but also accommodates volume change of Co_3_O_4_ during the charge–discharge cycling process. The electrode exhibits a high discharge capacity of 1352 mA h g^−1^ after 500 cycles at a constant current density of 0.2 A g^−1^. Additionally, the composites deliver a discharge capacity of 661 mA h g^−1^ with a small capacity decay of 0.078% per cycle at a high current density of 2 A g^−1^ after 500 cycles. This hierarchical porous structural design presents an effective strategy to develop a hybrid nanofiber for improving lithium ion storage.

## Abstract 2

Hierarchical porous CNFs/Co_3_O_4_ nanofiber is rationally designed and constructed as an anode for achieving high capacity and stable lithium ion batteries.

## Graphical abstract

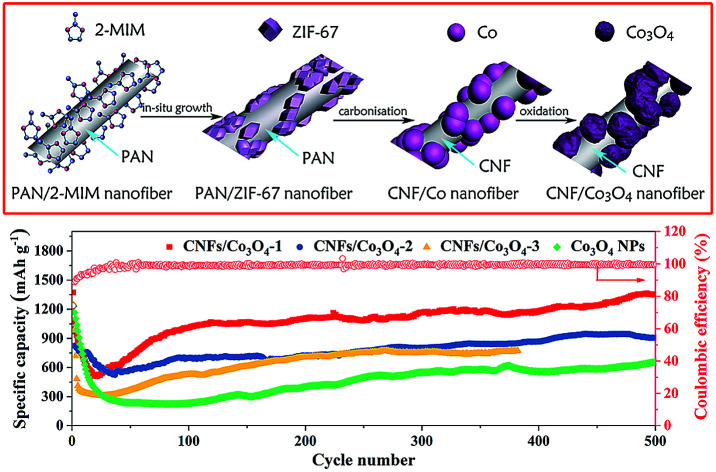

## Introduction

Energy and environmental crisis have triggered tremendous attention in developing efficient power systems.^1–4^ Meanwhile, as one of the most promising candidates for fast-developing technologies in electric energy storage, lithium ion batteries (LIBs) have been applied in electric vehicles and portable electronics owing to their impressive energy density, long lifespan and low cost.^5–7^ As a commercial anode material, graphite has a low theoretical capacity of 372 mA h g^−1^,^8^ which hinders the developments and applications of high energy density LIBs. Therefore, it is pivotal to design a novel anode material with higher capacity. As a representative of transition-metal oxides, Co_3_O_4_ possesses a high theoretical capacity of 890 mA h g^−1^.^9^ However, Co_3_O_4_ active material as an anode persistently suffers from unfortunate issues, including dramatic decreases in capacity and poor cycle performance induced by the structural instability and poor conductivity during cycling. To further get improvement in the performance for lithium ion storage, porous Co_3_O_4_ structures have been designed, such as porous hollow Co_3_O_4_ microspheres,^10^ porous Co_3_O_4_ cuboids,^11^ porous hollow Co_3_O_4_/N–C polyhedra,^12^ porous hollow Co_3_O_4_ parallelepipeds^6^*etc.* The porous architecture not only has a large specific surface area for tolerating the volume expansion of Co_3_O_4_ but also enriches transmission channels for lithium ions (Li^+^). The hierarchical porous structure helps excellent contact between the electrode and the electrolyte for producing a highly active interface area during charge–discharge cycling.^13–18^ As a late-model porous material, metal–organic frameworks (MOFs) have been extensively used as towardly templates to prepare porous metal-based compounds.^19–21^

As a typical MOFs, zeolitic imidazolate frameworks (ZIFs) with large surface area, high porosity and excellent chemical and thermal stability, have been applied in energy storage, gas adsorption and separation, luminescent sensors, and catalysis.^22–26^ Up to now, cobalt-based oxides with diverse morphologies have been synthesized by using ZIF-67 as a template, such as Fe_2_O_3_ nanotubes@Co_3_O_4_ composites,^27^ SnO_2_–Co_3_O_4_ nanofibers,^28^ Zn/Ni–Co-oxide,^7^ and Co_3_O_4_@Co_3_V_2_O_8_ nanoboxes^29^*etc.* Although great progresses have been made, the poor conductivity of ZIFs-derived Co_3_O_4_ still needs to be further improved.

One of the most emblematical carbon materials, carbon nanofibers (CNFs) possess the unique one-dimensional (1D) structure, favorable conductivity, excellent mechanical property, and chemical stability, which are beneficial for improving the electrochemical performance of LIBs.^30–33^ Electrospinning is a convenient, cheap and versatile method to successfully prepare 1D nanostructures with diversified diameters and morphologies.^34–37^ Consequently, a rational combination of involving CNFs obtained by electrospinning with the ZIF-derived transition metal oxide is able to construct excellent composite electrode materials to make up the deficiencies of single metal oxide electrode.

Herein, we have been successfully constructed hierarchical porous CNFs/Co_3_O_4_ composite nanofibrous materials. The ZIF-67 nanocrystalline have been grown on polyacrylonitrile/2-methylimidazole (PAN/2-MIM) nanofibers by *in situ* growth strategy. Afterwards, the hierarchical porous CNFs/Co_3_O_4_ composite materials were synthesized through a multi-step heat-treatment. The electrochemical properties of the prepared samples were systematically investigated. Encouragingly, the hierarchical porous CNFs/Co_3_O_4_ composite nanofibrous materials exhibit superior lithium storage performance, rate capability and remarkable cycling stability as an anode material for LIBs.

## Experimental section

### Chemicals

Polyacrylonitrile (PAN), cobalt nitrate hexahydrate (Co(NO_3_)_2_·6H_2_O), 2-methylimidazole (C_4_H_6_N_2_, 2-MIM), methanol (CH_3_OH), and *N*,*N*-dimethylformamide (DMF) were all purchased from Aladdin Chemical Reagent Co. All chemicals were of analytic grade and used without further purification.

### Preparation of PAN/2-MIM composite nanofibers

PAN/2-MIM composite nanofibers were obtained through an ordinary single-spinneret electrospinning technique. First of all, 1.0 g of PAN was dissolved in 9.5 g of DMF with continuously stirring at 60 °C for 4 h to form a homogenous solution. Then, 2.0 g of 2-MIM was added into the above solution at room temperature for 15 min under magnetic stirring. Subsequently, the obtained spinning solution was loaded into a 10 mL syringe, the distance between the plastic needle and collector (an aluminum foil) was 15 cm. A high voltage of 10 kV was supplied between the spinneret and the collector. Thus, PAN/2-MIM composite nanofibers were collected on the aluminum foil.

### Preparation of PAN/ZIF-67 composite nanofibers

PAN/ZIF-67 composite nanofibers were fabricated *via in situ* growth method. Co(NO_3_)_2_·6H_2_O was dissolved in 5 mL methanol to form a clear solution. Then the as-prepared PAN/2-MIM composite nanofibers (0.5 g) were soaked into the above solution at room temperature for 6 h. The molar ratios of 2-MIM to Co^2+^ were 4 : 0.25, 4 : 0.5, and 4 : 1, respectively. Hence, the products were respectively labeled as PAN/ZIF-67-1, PAN/ZIF-67-2, PAN/ZIF-67-3.

### Preparation of hierarchical porous CNFs/Co_3_O_4_ composite nanofibrous materials

The PAN/ZIF-67 composite nanofibers were stabilized at 250 °C for 90 min with a heating rate of 1 °C min^−1^ in air. Subsequently, the stabilized fibers were heated to 700 °C under argon flow for 2 h with a heating rate of 2 °C min^−1^. In the end, the sample was heated up to 350 °C at a ramp rate of 2 °C min^−1^ and then held for 30 min in air to fabricate the hierarchical porous CNFs/Co_3_O_4_ composite nanofibrous materials. According to the above molar ratios of 2-MIM to Co^2+^, the ultimate samples were respectively labeled as hierarchical porous CNFs/Co_3_O_4_-1, CNFs/Co_3_O_4_-2, and CNFs/Co_3_O_4_-3.

### Preparation of Co_3_O_4_ nanoparticles

In a typical process, 0.466 g of Co(NO_3_)_2_·6H_2_O and 1.050 g of 2-MIM were dissolved in 20 mL methanol, respectively. When the solution got clarified, the 2-MIM solution was poured into the pink solution with sostenuto stirring. The mixed solution was aged for 6 h at room temperature to obtain the ZIF-67. Subsequently, the purple precipitate was gathered through centrifugation three times, and then dried at 60 °C for 12 h in a vacuum oven. The Co_3_O_4_ nanoparticles were obtained *via* the decomposition of ZIF-67 in a muffle furnace under air, and the annealing temperature was 350 °C for 30 min at heating rate of 1 °C min^−1^.

### Characterization methods

The crystallographic information of the as-obtained samples was characterized by an X-ray powder diffractometer (XRD, Bruker, D8FOCUS). The morphologies and microstructures of the as-synthesized samples were investigated by transmission electron microscope (TEM, JEM-2100, JEOL) and scanning electron microscope (SEM, JSM-7800F, JEOL) with energy-dispersive X-ray spectrometer (EDX). The spectra of samples scope were measured with an ESCALAB 250 X-ray photoelectron spectrometer (XPS). N_2_ adsorption–desorption isotherms were tested by using an intelligent gravimetric analyzer Autosorb-iQ (Quantachrome). The content of the carbons was examined with a Thermogravimetric analyser (TA Instruments, SDT 2960) in air. The temperature was increased to heat the samples from room temperature to 950 °C at a rate of 20 °C min^−1^.

### Electrochemical measurements

To evaluate the electrochemical performances of the as-prepared samples, the CR2032 coin half-cells with a pure lithium foil as the counter electrode, Celgard 2320 as the separator, and 1 mol L^−1^ LiPF_6_ in ethylene carbonate/dimethyl carbonate (1 : 1 v/v) solution as the electrolyte were assembled in a glove box full of pure argon (H_2_O, O_2_ content < 1 ppm). The working electrodes were originated from a uniform slurry with a weight ratio of 80% active materials, 10% carbon black, and 10% polyvinylidene fluoride dissolved in *N*-methyl-2-pyrrolido. Then the slurry was coated on copper foil and dried in a vacuum oven at 120 °C overnight. The range of active material mass loading on the electrode was 0.74–0.87 mg cm^−2^ and the thickness of electrode film after drying was *ca.* 16 μm. Galvanostatic discharge–charge tests were carried out using a battery testing system (BTS-5 V/10 mA, Neware Technology Limited Corporation, China) with a cut-off potential of 0.01–3.0 V *versus* Li/Li^+^. The cyclic voltammetry (CV) measurements were performed on an electrochemical workstation (CHI-760D, Shanghai Chenhua Instrument Limited Corporation, China) with the voltage range of 0.01–3 V and a scan rate of 0.1 to 0.8 mV s^−1^. Electrochemical impedance spectroscope (EIS) was performed on an electrochemical workstation (CHI-760D) at open circuit voltage with amplitude of 5 mV in the frequency range from 1 MHz to 0.01 Hz.

## Results and discussion

### Formation mechanism of hierarchical porous CNFs/Co_3_O_4_ composite nanofibrous material

**Fig. 1:**
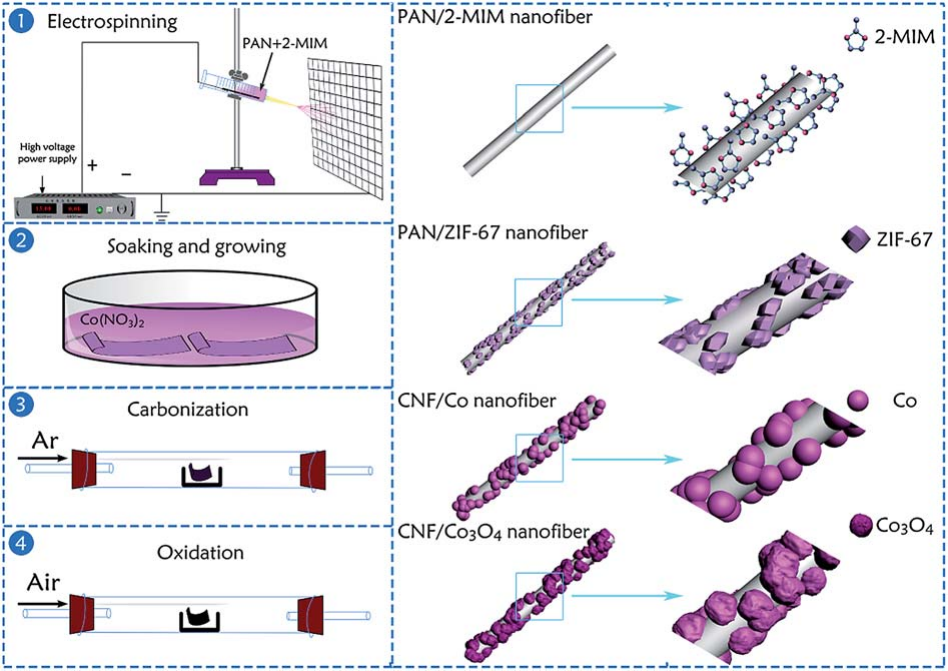
Schematic illustration of the formation process of hierarchical porous CNFs/Co_3_O_4_.

The manufacturing approach for the hierarchical porous CNFs/Co_3_O_4_ composite material is schematically described in Fig. 1. Firstly, the spinning solution containing PAN, 2-MIM and DMF was used for electrospinning to obtain the PAN/2-MIM composite nanofibers. On account of the strong complexation between 2-MIM and Co^2+^, when the PAN/2-MIM composite nanofibers were soaked into Co(NO_3_)_2_·6H_2_O methanol solution, the PAN/ZIF-67 composite nanofibers were formed. Meanwhile, some Co^2+^ diffused into PAN/ZIF-67 composite nanofibers to obtain ZIF-67 particles which were partly embedded in the fibers. Subsequently, the synthetized PAN/ZIF-67 composite nanofibers were transformed into CNFs/Co nanofibers *via* a heat-treatment in argon. In this atmosphere, not only the CNFs were generated on account of the carbonization of PAN nanofibers, but also the elemental Co nanoparticles were formed owing to the reduction of carbon.^33^ Conclusively, these Co nanocrystals were oxidized to Co_3_O_4_ through an annealing treatment in air and the hierarchical porous structure were formed due to the decomposition of 2-MIM during multi-step calcination process.

### Structures and morphology of hierarchical porous CNFs/Co_3_O_4_ composite nanofibrous material

**Fig. 2:**
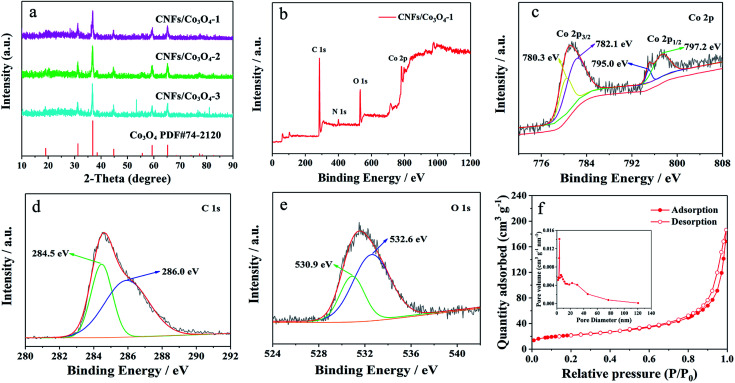
(a) XRD patterns of hierarchical porous CNFs/Co_3_O_4_ composite materials with different molar ratio of 2-MIM to Co^2+^. (b) XPS survey spectrum for the hierarchical porous CNFs/Co_3_O_4_-1 and the high-resolution spectra for (c) Co 2p, (d) C 1s and (e) O 1s. (f) Nitrogen adsorption–desorption isotherms of the hierarchical porous CNFs/Co_3_O_4_-1, inset: the corresponding pore size distribution curves.

The composition and crystal phase of hierarchical porous CNFs/Co_3_O_4_ with different molar ratios of 2-MIM and Co^2+^ are examined by X-ray diffraction (XRD). As revealed by Fig. 2a, the main diffraction peaks located at 18.99°, 37.27°, 36.84°, 44.80°, 59.35°, and 65.23° can be well indexed to Co_3_O_4_ (JCPDS card No. 74-2120). Upon increasing the content of Co^2+^, the strength of diffraction peaks gradually enhances. There are no obvious carbon peaks in XRD patterns, because carbon exists in the form of amorphous state. To examine the surface chemical information of the hierarchical porous CNFs/Co_3_O_4_-1, XPS is provided in Fig. 2b. The signals of C, O, Co and N can be observed, conforming that the hierarchical porous CNFs/Co_3_O_4_-1 is composed of carbon, oxygen, cobalt and nitrogen elements. The presence of nitrogen species in the conductive carbon backbone might accelerate electrode transfer and produce additional active sites to store lithium ions. As shown in Fig. 2c, the Co 2p spectrum could be deconvoluted into a pair of peaks at 782.1/797.2 eV and 780.3/795.0 eV after Shirley background subtraction and a Gaussian fitting treatment, corresponding to Co 2p_1/2_ and 2p_3/2_ of Co_3_O_4_,^9^ respectively. This result further confirms the formation of Co_3_O_4_. Furthermore, the C 1s spectrum (Fig. 2d) can be fitted into two peaks which are placed at 284.5 eV and 285.9 eV, assigning to C–C/C=C and C–O,^38,39^ respectively. Fig. 2e depicts the O 1s spectrum is decomposed into two peaks located at 530.9 eV and 532.6 eV, corresponding to Co–O and C–O,^9^ respectively. As shown in Fig. S1,† the TGA curves of the hierarchical porous CNFs/Co_3_O_4_-1, CNFs/Co_3_O_4_-2 and CNFs/Co_3_O_4_-3 obtained in air show that the content of C is 15.5%, 7.0%, and 6.7%, respectively.

The specific surface areas and porous natures of hierarchical porous CNFs/Co_3_O_4_-1, CNFs/Co_3_O_4_-2 and CNFs/Co_3_O_4_-3 are investigated by nitrogen adsorption–desorption isotherms. Fig. 2f presents the typical type IV curves, illustrating the existence of mesopores in the as-prepared hierarchical porous CNFs/Co_3_O_4_-1. The Brunauer–Emmett–Teller (BET) surface area of hierarchical porous CNFs/Co_3_O_4_-1 is 74.90 m^2^ g^−1^, which is higher than that of CNFs/Co_3_O_4_-2 (54.69 m^2^ g^−1^) and CNFs/Co_3_O_4_-3 (49.83 m^2^ g^−1^), respectively. The largest surface area of hierarchical porous CNFs/Co_3_O_4_-1 is attributed to the decomposition of 2-MIM during multi-step calcination. The molar ratios of 2-MIM to Co^2+^ were 4 : 0.25, 4 : 0.5, and 4 : 1, respectively. The content of 2-MIM in CNFs/Co_3_O_4_-1 is biggest among the three samples. Therefore, CNFs/Co_3_O_4_-1 possesses the highest surface area. In addition, the pore sizes of hierarchical porous CNFs/Co_3_O_4_-1 are respectively 3.88 nm, 6.44 nm, and 22.74 nm, and the total pore volume of hierarchical porous CNFs/Co_3_O_4_-1 is 0.03757 m^3^ g^−1^. Such distinctive porous structure with large surface area not only can provide enough active sites for the electrolyte contact but also supply enough space for the volume expansion of Co_3_O_4_ upon cycling.

**Fig. 3:**
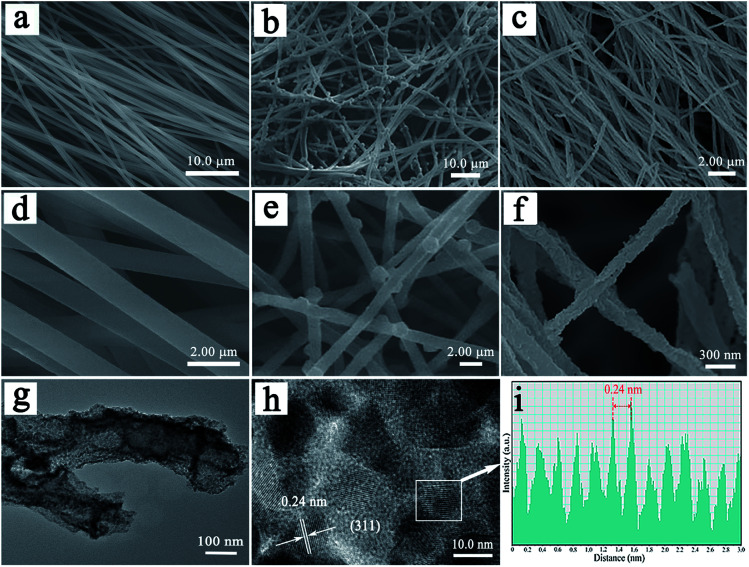
SEM images of (a) PAN/2-MIM composite nanofibers, (b) PAN/ZIF-67-1 composite nanofibers, and (c) hierarchical porous CNFs/Co_3_O_4_-1, (d–f) magnified view of the images of (a–c). (g) TEM and (h) HRTEM images of hierarchical porous CNFs/Co_3_O_4_-1, (i) the intensity plot of *d*-spacing for the (311) plane of Co_3_O_4_ in (h).

The morphologies of PAN/2-MIM composite nanofibers, PAN/ZIF-67, and hierarchical porous CNFs/Co_3_O_4_ are examined by SEM. In Fig. 3a and d, the SEM images demonstrate that the PAN/2-MIM composite nanofibers exhibit smooth surface and uniform diameter of *ca.* 1 μm. The SEM images of PAN/ZIF-67-1 are shown in Fig. 3b and e. Interestingly, it can be clearly seen that there are some ZIF-67 particles with rhombic dodecahedron shapes (with a diameter of 1 μm) directly formed on PAN/2-MIM composite nanofibers. The result is attributed to the fact that the coordination of Co^2+^ ions with 2-MIM to grow ZIF-67 not only on the surface of PAN/2-MIM composite nanofibers but also partially embed inside the composite nanofibers. Furthermore, the morphologies of PAN/ZIF-67-2 and PAN/ZIF-67-3 are similar to that of PAN/ZIF-67-1, as shown in Fig. S3a and S4a.† The images in Fig. 3c and f elucidate that the hierarchical porous CNFs/Co_3_O_4_-1 retains well the original fibered morphology of the PAN/ZIF-67 composite nanofibers. Furthermore, the resultant hybrid nanofiber displays an interconnected porous fiber architecture with a diameter distribution ranging from 150 nm to 250 nm. The evident diminution of diameter and formation of porous structure for hierarchical porous CNFs/Co_3_O_4_-1 is owing to the pyrocondensation of PAN and decomposition of 2-MIM during carbonization. Additionally, the ZIF-67 particles are converted into Co_3_O_4_ nanoparticles. It can be easily seen that the Co_3_O_4_ nanoparticles with small size distribute evenly on the surface of nanofibers. The morphologies of CNFs/Co_3_O_4_-2 and CNFs/Co_3_O_4_-3 are alike to that of hierarchical porous CNFs/Co_3_O_4_-1 in Fig. S3b and S4b.† Furthermore, Fig. S5† elucidates the EDX mapping images of hierarchical porous CNFs/Co_3_O_4_-1, CNFs/Co_3_O_4_-2, and CNFs/Co_3_O_4_-3, which display the uniform distribution of C, Co, and O elements. To further demonstrate the morphology of hierarchical porous CNFs/Co_3_O_4_-1, the TEM analysis is performed. Fig. 3g shows a typical porous nanofiber structure of hierarchical porous CNFs/Co_3_O_4_-1. In Fig. 3h and i, the high-resolution TEM (HRTEM) image reveals lattice fringes with a spacing of 0.24 nm, which is assigned to (311) plane of Co_3_O_4_. The hierarchical porous hybrid design provides a continuous porous conductive carbon backbone for sustaining structural stability and expediting lithium ion diffusion and electron transfer, and sufficient space to accommodate the volume change during charge–discharge process.

### Electrochemical performance

**Fig. 4:**
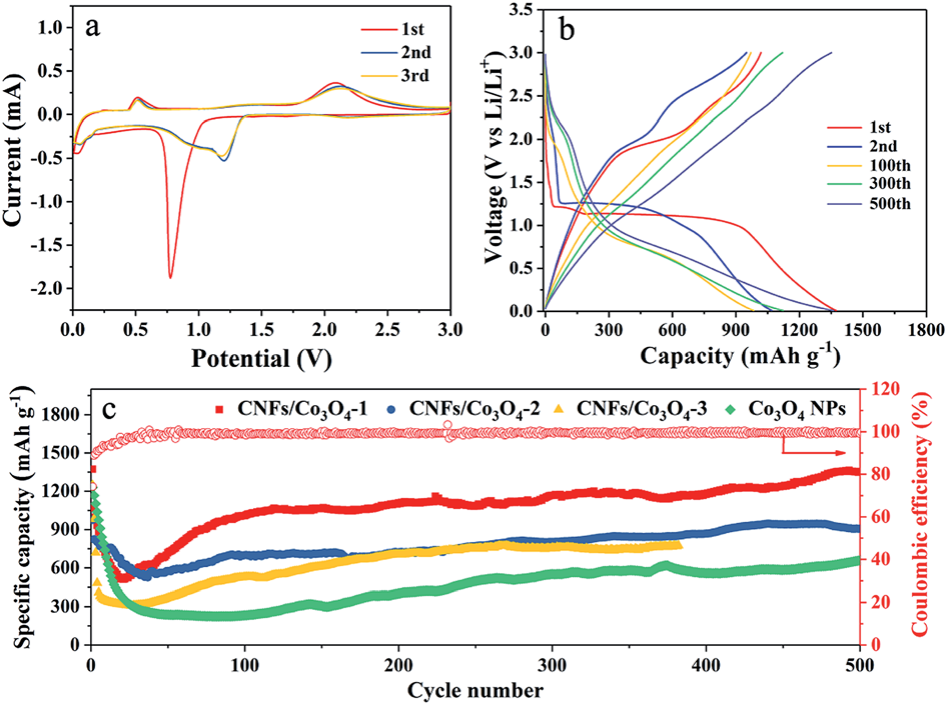
(a) CV curves of hierarchical porous CNFs/Co_3_O_4_-1 at a scan rate of 0.1 mV s^−1^ in the voltage range of 0.01–3.0 V *vs.* Li/Li^+^. (b) Galvanostatic discharge–charge voltage profiles of hierarchical porous CNFs/Co_3_O_4_-1 for the 1st, 2nd, 100th, 300th, and 500th cycles at a current rate of 0.2 A g^−1^ between 0.01 and 3.0 V. (c) Discharge−charge capacities of hierarchical porous CNFs/Co_3_O_4_-1, CNFs/Co_3_O_4_-2, CNFs/Co_3_O_4_-3, and Co_3_O_4_ and corresponding coulombic efficiency of hierarchical porous CNFs/Co_3_O_4_-1 at a current rate of 0.2 A g^−1^.

To examine the electrochemical properties of hierarchical porous CNFs/Co_3_O_4_-1 as an anode material for LIBs, the CV and galvanostatic charge–discharge measurements were carried out. Fig. 4a verifies the representative CV curves of hierarchical porous CNFs/Co_3_O_4_-1 at a scan rate of 0.1 mV s^−1^ in the potential window 0.01–3 V. There is a primary peak at ∼0.78 V in the first cathodic sweep, corresponding to the reduction of Co_3_O_4_ to metallic Co with the formation of a solid electrolyte interface (SEI) film.^40^ The intense cathodic peak shifts to 1.2 V during the following cycles, manifesting an irreversible transformation in the first cycle.^41^ Moreover, the peak at ∼2.1 V denotes the oxidation of Co to Co_3_O_4_ during the anodic process.^12^ In the subsequent cycles, the peaks are overlapped, implying the superior electrochemical reversibility of hierarchical porous CNFs/Co_3_O_4_-1. The involved electrochemical conversion reactions of hierarchical porous CNFs/Co_3_O_4_-1 can be depicted as follows: Co_3_O_4_ + 8Li^+^ + 8e^−^ ↔ 4Li_2_O + 3Co.

Fig. 4b presents the charge–discharge voltage profiles for the 1st, 2nd, 100th, 300th, and 500th cycles of the hierarchical porous CNFs/Co_3_O_4_-1 electrode at a constant current density of 0.2 A g^−1^ between 0.01 and 3.0 V. During the initial discharge process, there are a small flat plateau at 1.22 V and a long voltage plateau at 1.13 V. The discharge voltage plateau shifts upward to 1.26 V in the second discharge curve, perhaps attributed to the structural reorganization of the electrode materials in the first cycle, which is consistent with the CV results. The first charge and discharge capacities of hierarchical porous CNFs/Co_3_O_4_-1 are 1018 and 1373 mA h g^−1^, respectively, which yields the coulombic efficiency of 74.1%. The irreversible capacity might be ascribed to the irreversible decomposition of the electrolyte and the formation of the SEI film.^42^ Meanwhile, the 2nd, 100th, 300th, and 500th discharge capacities of the hierarchical porous CNFs/Co_3_O_4_-1 electrode are as high as 1069 mA h g^−1^, 984 mA h g^−1^, 1123 mA h g^−1^ and 1361 mA h g^−1^, which yield the coulombic efficiency of 88.8%, 98.6%, 99.2%, and 99.6%, respectively. The hierarchical porous CNFs/Co_3_O_4_-1 electrode exhibits a favourable reversible capacity.

Fig. 4c exhibits the cycling stabilities and coulombic efficiencies of hierarchical porous CNFs/Co_3_O_4_-1, CNFs/Co_3_O_4_-2, CNFs/Co_3_O_4_-3, and Co_3_O_4_ nanoparticles at a constant charge–discharge current density of 0.2 A g^−1^. The hierarchical porous CNFs/Co_3_O_4_-1 electrode delivers an initial capacity of 1373 mA h g^−1^. Although a decrease in specific capacity is observed during the first 24 cycles due to the formation of SEI film, the specific capacity gradually rises from 25th cycles through the lithiation-induced reactivation. It is attractive that the capacity can reach as high as 1352 mA h g^−1^ after 500 discharge–charge cycles. However, the capacities of CNFs/Co_3_O_4_-2, CNFs/Co_3_O_4_-3 and Co_3_O_4_ nanoparticles only maintain at 907 mA h g^−1^, 776 mA h g^−1^ and 659 mA h g^−1^ after 500, 380, and 500 cycles, respectively. Furthermore, it is also found that the hierarchical porous CNFs/Co_3_O_4_-1 exhibits more favourable cycling stability than others. The outstanding electrochemical performance can be attributed to the effect of hierarchical porous materials and the carbon content.^3,43^ To sum up, this performance is superior to that of many other cobalt oxides-based anodes, as summarized in Table S1.† The distinctive hierarchical porous structure and compositional features can provide adequate contact between the electrolyte and the electrode surface, which reduces the osmotic resistance of electrolyte, shortens the diffusion path of lithium ions, and buffers volume expansion of Co_3_O_4_ upon cycling. The comminution of active Co_3_O_4_ can be avoided due to implanting Co_3_O_4_ in the CNFs. Additionally, the CNFs not only maintain good integrity of the structure but also provide favorable conductivity, which makes the hierarchical porous CNFs/Co_3_O_4_ electrode suitable for cycling at high current density. To further confirm the structural stability of the designed electrode, the morphologies of hierarchical porous CNFs/Co_3_O_4_-1 before and after 150 cycles at a current density of 1 A g^−1^ are presented in Fig. S6.† It is seen that the morphology of designed electrodes are basically retained after 150 cycles in Fig. S6.†

**Fig. 5:**
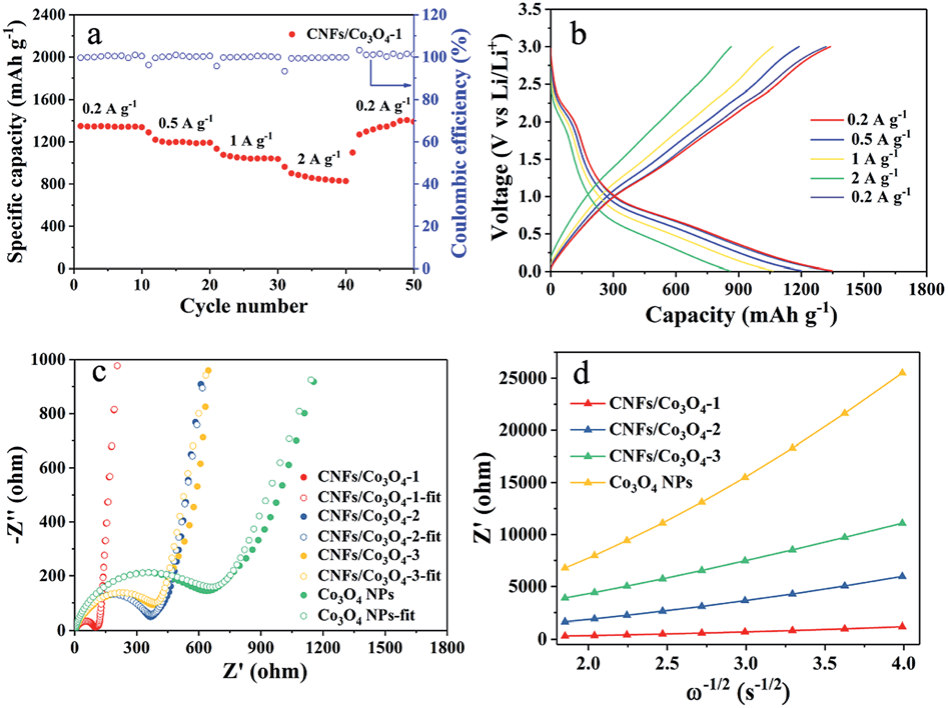
(a and b) Rate capabilities and galvanostatic discharge–charge voltage profiles of hierarchical porous CNFs/Co_3_O_4_-1 at a different current rate from 0.2 to 2 A g^−1^. (c) EIS spectra of hierarchical porous CNFs/Co_3_O_4_-1, CNFs/Co_3_O_4_-2, CNFs/Co_3_O_4_-3 and Co_3_O_4_ over the frequency ranging from 100 kHz to 0.01 Hz. (d) The real part of the complex impedance *vs. ω*^−1/2^ for these electrodes.

In order to further evaluate the superior electrochemical performance, the rate capability of hierarchical porous CNFs/Co_3_O_4_-1 has been explored based on the cycle at various current densities. Fig. 5a illustrates that the hierarchical porous CNFs/Co_3_O_4_-1 exhibits splendid capacity retention despite sustaining rapid changes in current density. The average discharge capacities of hierarchical porous CNFs/Co_3_O_4_-1 are 1345, 1207, and 1058 mA h g^−1^ at current densities of 0.2, 0.5, and 1 A g^−1^, severally. Even at a higher current density of 2 A g^−1^, the discharge capacity is as high as 867 mA h g^−1^. It is worth noting that the discharge capacity could still resume well to 1321 mA h g^−1^ when the current density returns to 0.2 A g^−1^. Notably, the specific capacity of hierarchical porous CNFs/Co_3_O_4_-1 is as high as 661 mA h g^−1^ with a coulombic efficiency of 100% after the 500 cycles at a current density of 2 A g^−1^ (Fig. S7†), demonstrating the preeminent specific capacity and cycling stability. As shown in Fig. 5b, the galvanostatic discharge–charge voltage profiles of hierarchical porous CNFs/Co_3_O_4_-1 at a different current rate from 0.2 to 2 A g^−1^ are similar, demonstrating that the electrode material presents excellent stability at high current densities. The eximious rate capability of hierarchical porous CNFs/Co_3_O_4_-1 can be ascribed to the improvement of kinetic process.

The EIS tests are conducted to evaluate the kinetics of hierarchical porous CNFs/Co_3_O_4_-1, CNFs/Co_3_O_4_-2, CNFs/Co_3_O_4_-3, and Co_3_O_4_ electrodes. As shown in Fig. 5c, each of Nyquist plots consists of a semicircle in the high frequency region and an oblique line in the low-frequency region. Furthermore, the intercept of the real axis represents the internal resistance (*R*_e_) and the diameter of the semicircle is related to the charge transfer resistance (*R*_ct_). The kinetic properties of hierarchical porous CNFs/Co_3_O_4_-1, CNFs/Co_3_O_4_-2, CNFs/Co_3_O_4_-3, and Co_3_O_4_ nanoparticles are analysed by ZSimWin software. Based on the equivalent circuit in Fig. S8,† these impedance data (Table S2†) are obtained. Fig. 5c expressly demonstrates the value of *R*_ct_ for the hierarchical porous CNFs/Co_3_O_4_-1 is 100.4 Ω, which exhibits a much smaller size of semicircle diameter than other samples. The slope of inclined straight line represents the Warburg coefficient that is associated with the diffusion rate of Li^+^. The smaller slope of the inclined straight line implies the faster diffusion of Li^+^. Fig. 5d distinctly depicts that the hierarchical porous CNFs/Co_3_O_4_-1 possesses smallest Warburg coefficient, indicating better diffusion capability of lithium ion. The EIS results of hierarchical porous CNFs/Co_3_O_4_-1, CNFs/Co_3_O_4_-2 and CNFs/Co_3_O_4_-3 after 150 charge–discharge cycles at a current density of 1 A g^−1^ are shown in Fig. S9.† It is easily seen that the *R*_ct_ values of the samples are reduced, and the *R*_ct_ of hierarchical porous CNFs/Co_3_O_4_-1 (56.96 Ω) is the smallest among the three samples. The *R*_ct_ decrement is probably due to the lattice expansion and surface activation of the electrodes, which promotes the charge transfer kinetics.^12^ The results further validate that the hierarchical porous CNFs/Co_3_O_4_-1 has a marked enhancement in ionic diffusion kinetics, which may be ascribed to follow aspects: (i) the plenty of pores on the fiber surface can shorten the diffusion path of Li^+^. (ii) CNFs with favorable conductivity can accelerate electron transfer, to induce fast electrochemical reaction.

**Fig. 6:**
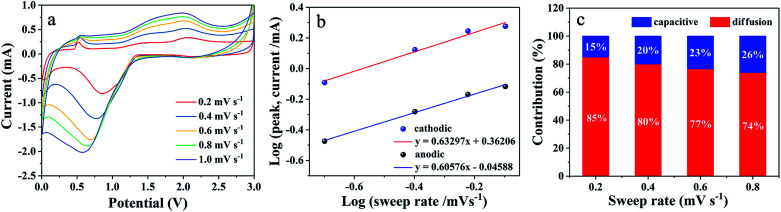
(a) CV curves of hierarchical porous CNFs/Co_3_O_4_-1 at different scan rates. (b) log(*i*) *versus* log(*v*) plots at different cathodic/anodic peaks. (c) Normalized contribution ratio of capacitance and diffusion at different scan rates.

To further expound the probable cause for the outstanding cycling and rate performance of hierarchical porous CNFs/Co_3_O_4_-1, the CV measurements at various scan rate from 0.2 to 0.8 mV s^−1^ are presented in Fig. 6. Fig. 6a depicts the shape of peaks is similar and gradually broaden with increasing scan rate. There is an interrelation between the measured current (*i*) and scan rate (*ν*): *i* = *aν*^*b*^, where *a* and *b* are adjustable constants.^44,45^ It indicates that the current can be controlled by diffusion-controlled insertion process (*b* value close to 0.5) or surface-induced capacitance process (*b* value close to 1), respectively.^46^ The diffusion-controlled insertion process is propitious to fast charge–discharge by multiple electron involved redox reaction. However, the surface-induced capacitance process is conducive to high capacity by ionic adsorption on the near surface of the active material.^47,48^ The value of *b* can be determined *via* the slope ratio of the log *i*–log *ν* plot. Fig. 6b illustrates that the *b* values of the cathodic and anodic peaks are 0.63 and 0.60, demonstrating that the kinetics are mainly diffusion-controlled insertion process. The equation *i* = *k*_1_*ν* + *k*_2_*ν*^1/2^ is used to further analyze the diffusion-controlled contribution, here, *k*_1_*ν* and *k*_2_*ν*^1/2^ correspond to surface-induced capacitance and diffusion-controlled contribution, respectively. Fig. 6c clearly exhibits the values of the diffusion contribution are 85%, 80%, 77%, and 74% at scan rates of 0.2, 0.4, 0.6, and 0.8 mV s^−1^, respectively. This result indicates that the diffusion-controlled contribution accounts for majority of the whole kinetics process and surface-induced capacitance provides extra capacity. The rapid lithium ion diffusion insertion and additional surface-induced capacitance can be attributed to the hierarchical porous structure and hybrid features of the hierarchical porous CNFs/Co_3_O_4_-1 electrode, to achieve the lithium storage performance and high-rate capability.

## Conclusions

In summary, the hierarchical porous CNFs/Co_3_O_4_ electrode materials have been successfully constructed and manifested excellent electrochemical performance. The hierarchical porous structure can efficiently shorten the diffusion path of lithium ions and accommodate the volume change of Co_3_O_4_ during cycling. More significantly, the CNFs are beneficial for improving the electronic conductivity and preserving the stability of the electrodes. The hierarchical porous CNFs/Co_3_O_4_-1 electrode delivers notable capacity, superior rate capacity, and excellent cycling performance. The hierarchical porous CNFs/Co_3_O_4_-1 electrode exhibits predominant capacity of 1352 and 661 mA h g^−1^ at 0.2 and 2 A g^−1^ up to 500 cycles, respectively. In particular, the electrode demonstrates a small capacity decay of only 0.078% per cycle at a high current density of 2 A g^−1^ after 500 cycles, implying the superior stability of hierarchical porous CNFs/Co_3_O_4_-1 electrode materials. Moreover, this synthetic strategy paves a new way to fabricate porous hybrid material for higher capacity LIB.

## Conflicts of interest

There are no conflicts to declare.

## Supplementary Material

RA-008-C8RA06307A-s001
