# Supplementary material for: Rationally designed hierarchical porous CNFs/Co3O4 nanofiber-based anode for realizing high lithium ion storage
Source: RSC Adv. 2018 Aug 31;8(54):30794–801. doi: 10.1039/c8ra06307a (PMC9085501; doi:10.1039/c8ra06307a)
Supplement: RA-008-C8RA06307A-s001 [file RA-008-C8RA06307A-s001.pdf]

Electronic Supplementary Material (ESI) for RSC Advances.
This journal is © The Royal Society of Chemistry 2018

Electronic Supplementary Information (ESI)

Rationally Designed Hierarchical Porous CNFs/Co_3O_4 Nanofiber-based Anode for Realizing High Lithium Ion Storage

He Wang, Yan Song, Yanwei Li, Mengwei Wang, Qianli Ma, Wensheng Yu, Dan Li, Xiangting Dong,* Jinxian Wang, Guixia Liu

School of Chemistry and Environmental Engineering, Changchun University of Science and Technology, Changchun 130022, China. E-mail: xtdong@cust.edu.cn Fax: 86-0431-85383815 Tel.: 86-0431-85582574

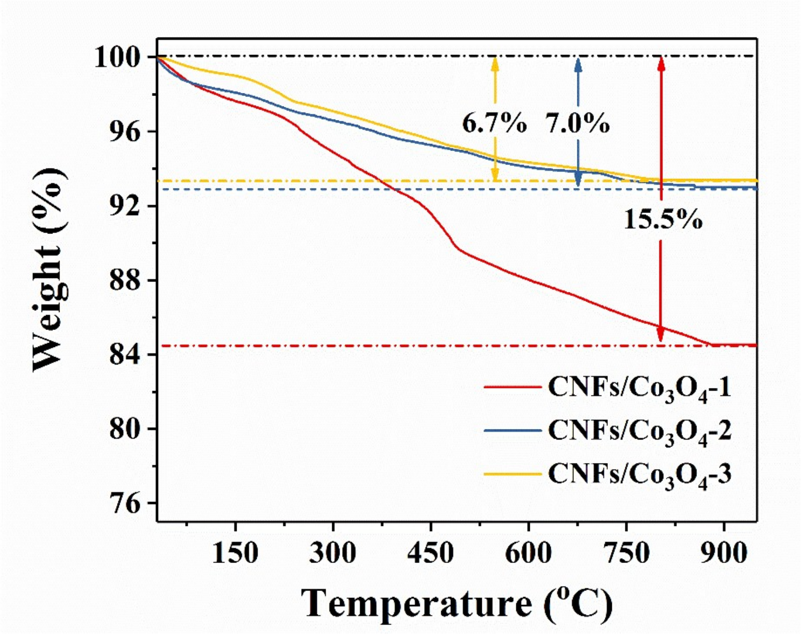

Fig. S1 TGA curves for the (a) hierarchical porous CNFs/Co_3O_4-1, (b) CNFs/Co_3O_4-2 and (c) CNFs/Co_3O_4-3.

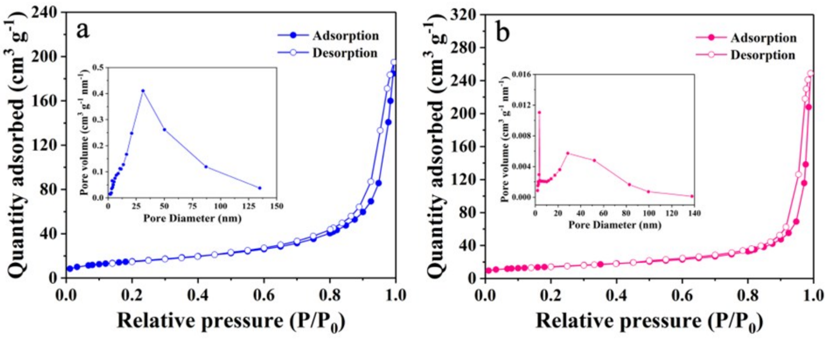

Fig. S2 Nitrogen adsorption-desorption isotherms of the (a) CNFs/Co_3O_4-2, and (b) CNFs/Co_3O_4-3 inset: the corresponding pore size distribution curves, respectively.

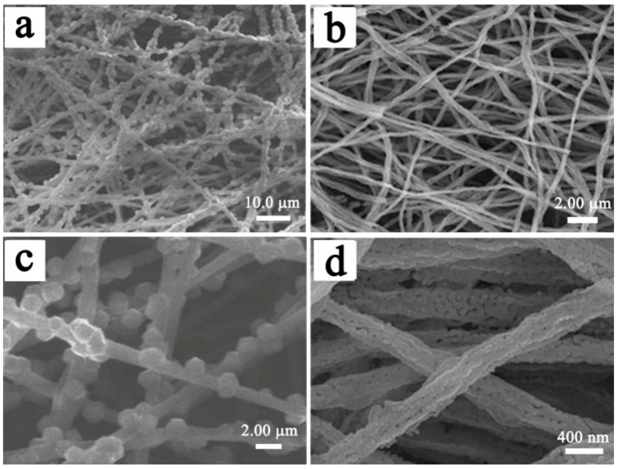

Fig. S3 SEM images of (a) PAN/ZIF-67-2 composite nanofibers, and (b) CNFs/Co_3O_4-2, (c, d) magnified view of the images of (a, b).

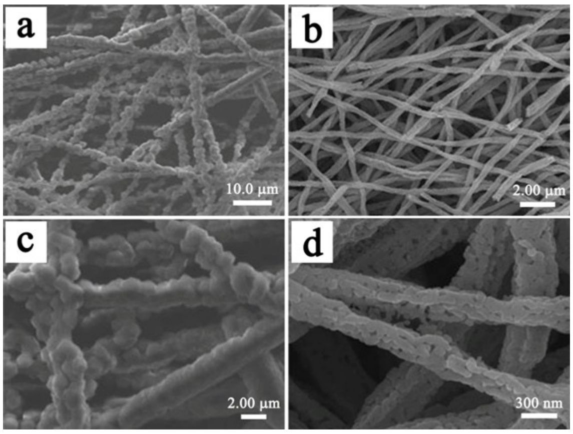

Fig. S4 SEM images of (a) PAN/ZIF-67-3 composite nanofibers, and (b) CNFs/Co_3O_4-3, (c, d) magnified view of the images of (a, b).

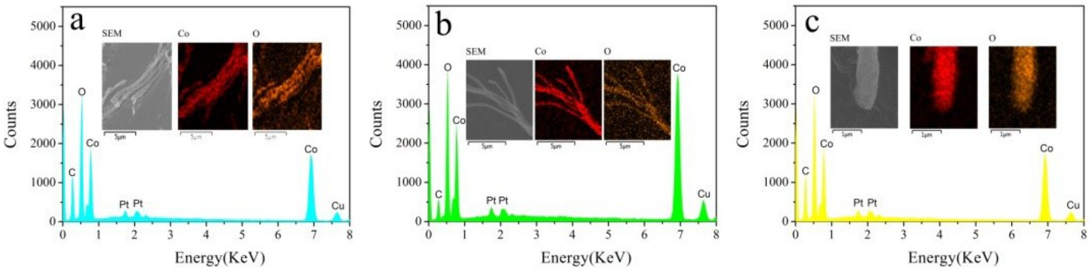

Fig. S5 EDX mapping images of hierarchical porous CNFs/Co_3O_4-1, CNFs/Co_3O_4-2 and CNFs/Co_3O_4-3.

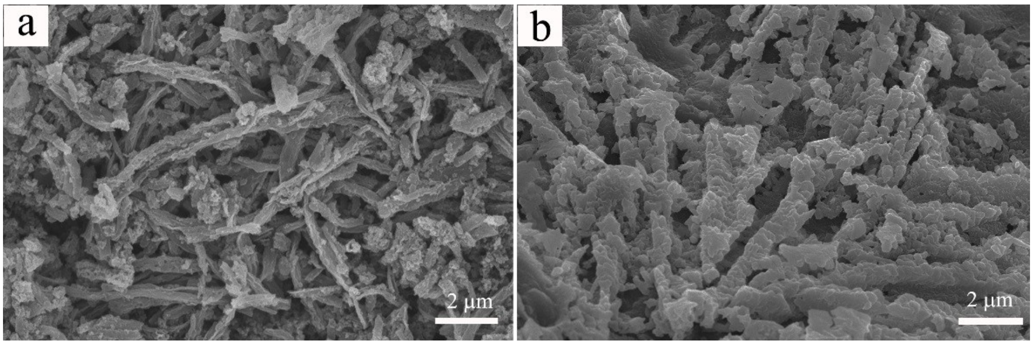

Fig. S6 SEM images of hierarchical porous CNFs/Co_3O_4-1 (a) before and (b) after 150 cycles at a current density of 1 A g^{-1}.

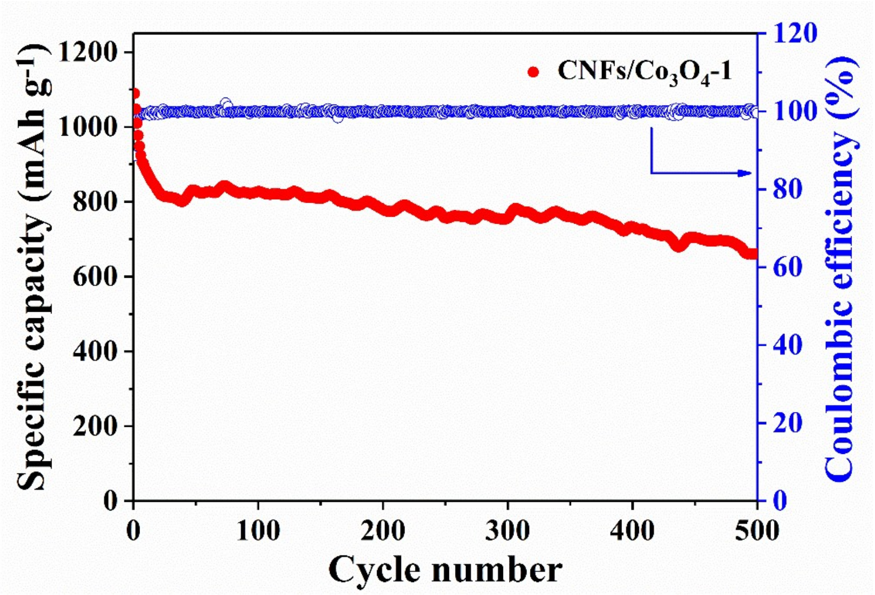

Fig. S7 Discharge−charge capacity and Coulombic efficiency of hierarchical porous CNFs/Co_3O_4-1 at a current density of 2 A g^{-1}.

Table S1 Comparison of lithium storage performance for the hierarchical porous CNFs/Co_3O_4-1 with other Co_3O_4-based electrodes.

Materials	Specific capacity	Cycle number	Ref.
Hierarchical porous CNFs/Co_3O_4-1	1352 mAh g^{-1} at 0.2A g^{-1}<br>661 mAh g^{-1} at 2A g^{-1}	500	This work
Co_3O_4@nitrogen-doped Grapheme network	995 mAh g^{-1} at 0.2A g^{-1}<br>676 mAh g^{-1} at 1A g^{-1}	100<br>400	1
Porous Co_3O_4 hollow Tetrahedral	1196 mAh g^{-1} at 0.05A g^{-1}<br>1052 mAh g^{-1} at 0.2 A g^{-1}	60	2
Graphene-embedded Co_3O_4 rose-spheres	990.8 mAh g^{-1} at 0.1 C (0.09 A g^{-1})<br>655.9 mAh g^{-1} at 2 C (1.8 A g^{-1})	200	3
Porous hollow Co_3O_4 parallelepipeds	1115 mAh g^{-1} at 0.1A g^{-1}	50	4
Porous N-doped carbon coated Co_3O_4 fish-scale	612 mAh g^{-1} at 1 A g^{-1}	500	5
Hierarchical three-dimensional flower-like Co_3O_4	1588 mAh g^{-1} at 0.1 A g^{-1}<br>1051 mAh g^{-1} at 1.12 C (1 A g^{-1})	100<br>800	6
Snowflake-shaped Co_3O_4	1044 mAh g^{-1} at 0.5 A g^{-1}<br>1051 mAh g^{-1} at 1 A g^{-1}<br>939 mAh g^{-1} at 2 A g^{-1}	100	7
Porous hollow Co_3O_4 microspheres	1012.7 mAh g^{-1} at 0.2 C (0.18 A g^{-1})<br>881.3 mAh g^{-1} at 2 C (1.8 A g^{-1})	100<br>300	8
Hierarchically structured Co_3O_4 @carbon porous fibers	1110 mAh g^{-1} at 0.1A g^{-1}<br>647 mAh g^{-1} at 1 A g^{-1}	200<br>700	9
Porous starfish-like Co_3O_4@nitrogen-doped carbon	795 mAh g^{-1} at 0.5A g^{-1}	500	10
Porous Co_3O_4 cubes@graphene	980 mAh g^{-1} at 0.2A g^{-1}	80	11
MWCNTs/Co_3O_4	813 mAh g^{-1} at 0.1A g^{-1}	100	12
Mesoporous perforated Co_3O_4 nanoparticles with a thin carbon layer	1115.1 mAh g^{-1} at 0.2 C (0.18 A g^{-1})	100	13

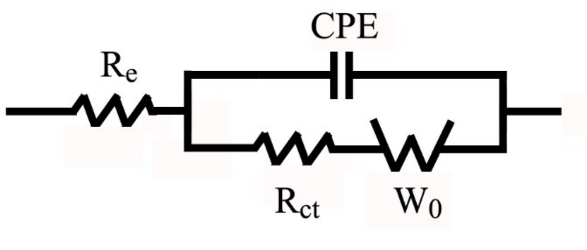

Fig. S8 Randles equivalent circuit.

Table S2 Fitted EIS results of the hierarchical porous CNFs/Co_3O_4-1, CNFs/Co_3O_4-2, CNFs/Co_3O_4-3 and Co_3O_4 NPs electrode.

	Hierarchical porous CNFs/Co_3O_4-1	Hierarchical porous CNFs/Co_3O_4-2	Hierarchical porous CNFs/Co_3O_4-3	Co_3O_4 NPs
R_e (Ω)	4.182	4.847	5.474	5.479
R_{ct} (Ω)	100.27	369.86	390.14	652.99

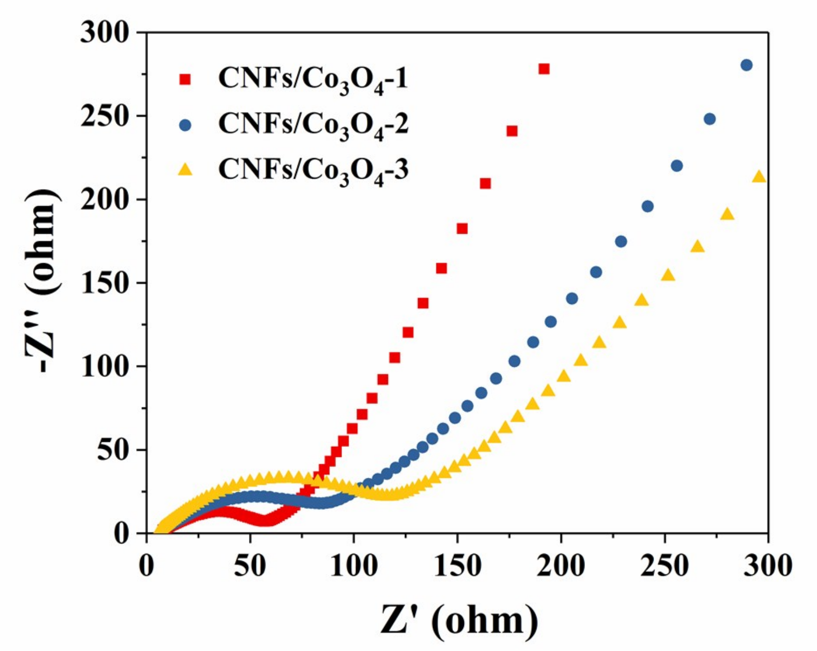

Fig. S9 EIS spectra of hierarchical porous CNFs/Co_3O_4-1, CNFs/Co_3O_4-2 and CNFs/Co_3O_4-3 electrode after 150 cycles at current of 1 A g^{-1}.

References

1 Z.-Y. Sui, P.-Y. Zhang, M.-Y. Xu, Y.-W. Liu, Z.-X. Wei and B.-H. Han, *ACS Appl. Mater. Interfaces*, 2017, **9**, 43171-43178.

2 D. Tian, X.-L. Zhou, Y.-H. Zhang, Z. Zhou and X.-H. Bu, *Inorg. Chem.*, 2015, **54**, 8159-8161.

3 M. Jing, M. Zhou, G. Li, Z. Chen, W. Xu, X. Chen and Z. Hou, *ACS Appl. Mater. Interfaces*, 2017, **9**, 9662-9668.

4 Y. Han, M. Zhao, L. Dong, J. Feng, Y. Wang, D. Li and X. Li, *J. Mater. Chem. A*, 2015, **3**, 22542-22546.

5 X. Han, W.-M. Chen, X. Han, Y.-Z. Tan and D. Sun, *J. Mater. Chem. A*, 2016, **4**, 13040-13045.

6 W. Cao, W. Wang, H. Shi, J. Wang, M. Cao, Y. Liang and M. Zhu, *Nano Res.*, 2018, **11**, 1437-1446.

7 B. Wang, X.-Y. Lu and Y. Tang, *J. Mater. Chem. A*, 2015, **3**, 9689-9699.

8 H. Du, K. Huang, M. Li, Y. Xia, Y. Sun, M. Yu and B. Geng, *Nano Res.*, 2018, **11**, 1490-1499.

9 C.-L. Zhang, B.-R. Lu, F.-H. Cao, Z.-L. Yu, H.-P. Cong and S.-H. Yu, *J. Mater. Chem. A*, 2018, **6**, 12962-12968.

10 Y. Sun, F. Huang, S. Li, Y. Shen and A. Xie, *Nano Res.*, 2017, **10**, 3457-3467.

11 H. Geng, Y. Guo, X. Ding, H. Wang, Y. Zhang, X. Wu, J. Jiang, J. Zheng, Y. Yang and H. Gu, *Nanoscale*, 2016, **8**, 7688-7694.

12 G. Huang, F. Zhang, X. Du, Y. Qin, D. Yin and L. Wang, *ACS Nano*, 2015, **9**, 1592-1599.

13 J. S. Park, D. O. Shin, C. S. Lee, Y.-G. Lee, J. Y. Kim, K. M. Kim and K. Shin, *Electrochimica Acta*, 2018, **264**, 376-385.
